# Supplementary material for: Factors associated with undernutrition among pregnant women in Haramaya district, Eastern Ethiopia: A community-based study
Source: PLoS One. 2023 Mar 9;18(3):e0282641. doi: 10.1371/journal.pone.0282641 (PMC9997975; doi:10.1371/journal.pone.0282641)
Supplement: Attachment — Submitted filename: 1 Review Reports for PLOS ONE.docx [file pone.0282641.s001.docx]

**Review Reports to the authors**

Manuscript Id: PONE-D-21-17941
Title: Determinants of Under-nutrition Among Pregnant Women in Haramaya District, Eastern Ethiopia

**Comments**

*While the title and the objectives talk about determinants of under-nutrition, the authors used a cross sectional study and reported only factors associated with under-nutrition, not the determinants. The authors should have used case control study. You have to modify the title as “Factors associated with under-nutrition among Pregnant Women in Haramaya District, eastern Ethiopia”, or otherwise the evidence presented doesn’t match your title and objectives.

*I suggest to write “Among” as ‘among’ and “Eastern” as ‘eastern’ in your revised title

You used different font styles for similar headings and sub-headings. Please check and revise it.

*The manuscript is full of grammatical and editorial errors. Please go through it line by line (thoroughly) and revise it, better checked by language professionals. Some of such errors are indicated below.

**Abstract**

Methods: - sectional study design was….

*But, what is sectional study design? Please make it clear.

- Data was collected with interviewers-administered questionnaires by well-trained health professionals.

*Make it data were collected with…. And this is also same under method section.

*well trained health professionals….who are these, HDSS staff? What do you mean by well trained? Please clearly indicate what their profession is, their level of education is and how their training was for this data collection. Describe these details under your methods section.

Results: line 2- “Under-nutrition” is written in different font from others.

-Line 3 Breakfast should be written as ‘breakfast’

Conclusion: **-**The the present study“

*What is “the the”? Please edit it.

- High prevalence “was observed on” women who “reported chat less than four times”

* was observed on should be written as “was found among”

*reported chat less than four times should be “ reported to chew Khat less than four times”

-Nutrition policy, programs and interventions….

* This is too general and ambiguous conclusion. Based on your findings specify to the point and indicate which policy, program and intervention should do what to improve which problem.

**Introduction**

*This section is not well written since doesn’t indicate the picture of maternal under-nutrition during pregnancy from global, regional, national and local contexts.

* The literatures are not well searched, and hence the introduction is too shallow. So many other related articles were missed.

*What is the importance of this study since the factors identified were already well known as you indicated under paragraph three? What is special now in this study? Can’t we use studies from other settings in Ethiopia for eastern Ethiopian regions?

-Line 2 ….affecting the healthy of women both in developed and developing countries, where, more than…

* write it as ‘affecting health of women both in developed and developing countries where more than…

Line 3…Moreover, under-nutrition occurred due to the…

*Write it as ‘Moreover, under-nutrition occurs due to the….

-Paragraph 4 line 2 ….and s nutritional status among

* re-write it as … and nutritional status among

**Methods**

*Chat has to be written as ‘Khat’, revise it accordingly throughout the document

*You excluded all pregnant women with reported acute and chronic illnesses. How trustable this reported illness is? And what number or how many percent of the women were excluded with these criteria?

-The sample size required and adequate for estimating the determinants of dietary practices of pregnant women was computed….

*Are you calculating for determinants of dietary practice or under nutrition? Be consistent, clear and to the point in line with your objectives.

*For sample size determination you just stated of using single and double population proportions. Would you indicate the double proportion formula and calculations used? *How many variables were used to try calculating your sample size using double proportion formula? Please indicate these as well.

* You described the role of agro-ecolocolgical difference on under-nutrition, but used study from Gumay district for sample size determination. Why? Any effect it might pose?

* Also mention names of those kebeles (with their total sample allocated), you used under the DSS and techniques followed using a simple diagrammatic presentation.

-After translation, it’s the consistency	* write it as “its consistency”

* Indicate/cite literatures you used for developing your questionnaire

- Hemoglobin analysis was carried out in the health post located in each Kebele by laboratory technologists.

* How was this practical? You were collecting data home to home but hemoglobin at health center? Were you calling each women to health center? What is the need to go to health center since you used Hemocue?

-Hemoglobin level was adjusted for altitude before the data were entered.

*How was the adjustment done- any formula? What about adjustment for other factors- did you considered any? Indicate how you classified anemia under analysis part.

* standard operating procedures (SOPs)….indicate these under the procedures….only SOP you used.

* How was anthropometric measurement standardized? Any TEM (technical error of measurements) done? Try to answer this in relation to the data collectors training, profession and how were they trained as raised above?

* under analysis- you still stated “to identify determinants of under-nutrition”….you rather should say factors associated with under-nutrition, not determinants…..And you may also need to re-consider your method of analysis appropriate to this…..logistic regression is one.

* What about those women under age regarding consent to participate? You had 24 women under the age of 18 years.

**Results**

*How do you see 100% response rate in a community based study like yours?

*Table 1- would you classify and present women’s education similar to husbands’ case?

*How do you see the wealth index vs women’s and husband’s occupation? Mismatch?

* Table 2: nutritional status ….write it as under-nutrition and normal….check typos errors in the table and also do not mix acute…make it consistent from title to conclusion.

* Chat chewing….make it “Khat”

* Table 3…factors determinants of under-nutrition…..vs the sub-title factors associated with??? And also vs manuscript title, check it again. I suggest this analysis method to be changed as indicated above.

* And also try to present some of your data with other data presentation techniques than table…

**Discussion**

*This section is very shallow. You are not supposed just to say similarity and difference ,rather explain what the public health implication of each finding is and interprete.

E.g. You put “This might be the gio-ecological variation of study set up, methodology used, where some of them were conducted at facility level which does not really show the real magnitude of the problem as of community level”.

*You have to tell the readers how geo-ecological variation matters, what method difference brought difference in the findings,,,,etc?

*Re-look at the strengths and limitations you put…a lot of limitations with less strengths.

**Conclusion
 ***How would you comment on wealth status and high under-nutrition in your findings?

*Check comments to the comments to conclusion under abstract

* And re-look at your recommendations regarding anemia and Iron folic acid supplementation.

**Consent for publication**

-Not applicable

*Authors should agree and give their consent for the publication of this paper…check that.

**Reference**

*Check other references are exhaustively searched and included. Reference style also needs some editorial corrections in line with journal requirements.

**The end!**
