# Supplementary material for: Factors associated with undernutrition among pregnant women in Haramaya district, Eastern Ethiopia: A community-based study
Source: PLoS One. 2023 Mar 9;18(3):e0282641. doi: 10.1371/journal.pone.0282641 (PMC9997975; doi:10.1371/journal.pone.0282641)
Supplement: Attachment — Submitted filename: Authors response to reviewers comments Final docx.docx [file pone.0282641.s002.docx]

PONE-D-21-17941

Determinants of Under-nutrition Among Pregnant Women in Haramaya District, Eastern Ethiopia

PLOS ONE

Thomas Phillips, PhD
Staff Editor
PLOS ONE,

First of all we would like to acknowledge you for your constructive and valuable comments and helpful
suggestions that helped us to improve and enrich our manuscript. Here under following editors comment and each reviewer’s we have pointed out how authors incorporated your valuable comments, suggestions and concerns one by one. On the following pages, you will find our response to the editor and reviewer comments. On behalf of my co-authors, I thank you for your consideration of this re-submission.

Meseret Belete Fite, meseretphd2014@gmail.com (correspondence author)

Editor comments and authors’ response

Thank you for submitting your manuscript to PLOS ONE. After careful consideration, we feel that it has merit but does not fully meet PLOS ONE’s publication criteria as it currently stands. Therefore, we invite you to submit a revised version of the manuscript that addresses the points raised during the review process.

The manuscript has been evaluated by two reviewers, and their comments are available below.The reviewers have raised a number of major concerns. They request improvements to the reporting of methodological aspects of the study and more information on how the data collection was completed.

The reviewers also note concerns about the statistical analyses presented and request re-analyses be completed. The reviewers suggest certain references be added to the text. While you are welcome to do so if you feel they are good suggestions you are not obligated to do so.

Could you please carefully revise the manuscript to address all comments raised?

Please submit your revised manuscript by Apr 29 2022 11:59PM. If you will need more time than this to complete your revisions, please reply to this message or contact the journal office at [plosone@plos.org](mailto:plosone@plos.org). When you're ready to submit your revision, log on to https://www.editorialmanager.com/pone/ and select the 'Submissions Needing Revision' folder to locate your manuscript file.

Please include the following items when submitting your revised manuscript:

- A rebuttal letter that responds to each point raised by the academic editor and reviewer(s). You should upload this letter as a separate file labeled 'Response to Reviewers'.
- A marked-up copy of your manuscript that highlights changes made to the original version. You should upload this as a separate file labeled 'Revised Manuscript with Track Changes'.
- An unmarked version of your revised paper without tracked changes. You should upload this as a separate file labeled 'Manuscript'.

If you would like to make changes to your financial disclosure, please include your updated statement in your cover letter. Guidelines for resubmitting your figure files are available below the reviewer comments at the end of this letter.

If applicable, we recommend that you deposit your laboratory protocols in protocols.io to enhance the reproducibility of your results. Protocols.io assigns your protocol its own identifier (DOI) so that it can be cited independently in the future. For instructions see: <https://journals.plos.org/plosone/s/submission-guidelines#loc-laboratory-protocols>. Additionally, PLOS ONE offers an option for publishing peer-reviewed Lab Protocol articles, which describe protocols hosted on protocols.io. Read more information on sharing protocols at <https://plos.org/protocols?utm_medium=editorial-email&utm_source=authorletters&utm_campaign=protocols>.

Thank you a lot for your substantial effort. Really this comment is helpful to enrich the manuscript. Authors get your concern and critically looked your comment

Kind regards,

Journal Requirements:

When submitting your revision, we need you to address these additional requirements.

1. Please ensure that your manuscript meets PLOS ONE's style requirements, including those for file naming. The PLOS ONE style templates can be found at 
https://journals.plos.org/plosone/s/file?id=wjVg/PLOSOne_formatting_sample_main_body.pdf and 
https://journals.plos.org/plosone/s/file?id=ba62/PLOSOne_formatting_sample_title_authors_affiliations.pdf

2. Please include additional information regarding the survey or questionnaire used in the study and ensure that you have provided sufficient details that others could replicate the analyses. For instance, if you developed a questionnaire as part of this study and it is not under a copyright more restrictive than CC-BY, please include a copy, in both the original language and English, as Supporting Information. If the original language is written in non-Latin characters, for example Amharic, Chinese, or Korean, please use a file format that ensures these characters are visible.

3. Please state whether you validated the questionnaire prior to testing on study participants. Please provide details regarding the validation group within the methods section.

4. Thank you for stating the following in the Acknowledgments Section of your manuscript:

The authors would like express sincere appreciation to Haramaya University for funding of this study.

Please note that funding information should not appear in the Acknowledgments section or other areas of your manuscript. We will only publish funding information present in the Funding Statement section of the online submission form. 
Please remove any funding-related text from the manuscript and let us know how you would like to update your Funding Statement. Currently, your Funding Statement reads as follows:

This study was fully funded by Haramaya University after the proposal has been defended. The funder has no role in conception, design of the study, statistical analysis, result interpretation and in writing up the manuscript. The funding institution has no role in the publication consent or approval.

Please include your amended statements within your cover letter; we will change the online submission form on your behalf.

5. Thank you for submitting the above manuscript to PLOS ONE. During our internal evaluation of the manuscript, we found significant text overlap between your submission and the following previously published works, some of which you are an author.

- https://www.hindawi.com/journals/aph/2018/1350195/
- https://www.dovepress.com/metabolic-syndrome-among-working-adults-in-eastern-ethiopia-peer-reviewed-fulltext-article-DMSO
- https://journals.plos.org/plosone/article?id=10.1371/journal.pone.0247085
- https://link.springer.com/article/10.1186/s40748-018-0087-z

We would like to make you aware that copying extracts from previous publications, especially outside the methods section, word-for-word is unacceptable. In addition, the reproduction of text from published reports has implications for the copyright that may apply to the publications.

Please revise the manuscript to rephrase the duplicated text, cite your sources, and provide details as to how the current manuscript advances on previous work. Please note that further consideration is dependent on the submission of a manuscript that addresses these concerns about the overlap in text with published work.

We will carefully review your manuscript upon resubmission, so please ensure that your revision is thorough.
[Note: HTML markup is below. Please do not edit.]

Reviewers' comments:

Reviewer's Responses to Questions

**Comments to the Author**

1. Is the manuscript technically sound, and do the data support the conclusions?

The manuscript must describe a technically sound piece of scientific research with data that supports the conclusions. Experiments must have been conducted rigorously, with appropriate controls, replication, and sample sizes. The conclusions must be drawn appropriately based on the data presented.

Reviewer #1: Yes

Reviewer #2: Partly

2. Has the statistical analysis been performed appropriately and rigorously?

Reviewer #1: Yes

Reviewer #2: No

3. Have the authors made all data underlying the findings in their manuscript fully available?

The [PLOS Data policy](http://www.plosone.org/static/policies.action#sharing) requires authors to make all data underlying the findings described in their manuscript fully available without restriction, with rare exception (please refer to the Data Availability Statement in the manuscript PDF file). The data should be provided as part of the manuscript or its supporting information, or deposited to a public repository. For example, in addition to summary statistics, the data points behind means, medians and variance measures should be available. If there are restrictions on publicly sharing data—e.g. participant privacy or use of data from a third party—those must be specified.

Reviewer #1: Yes

Reviewer #2: No

4. Is the manuscript presented in an intelligible fashion and written in standard English?

PLOS ONE does not copyedit accepted manuscripts, so the language in submitted articles must be clear, correct, and unambiguous. Any typographical or grammatical errors should be corrected at revision, so please note any specific errors here.

Reviewer #1: No

Reviewer #2: No

**Reviewer's comments and authors’ response**

5. Review Comments to the Author

Please use the space provided to explain your answers to the questions above. You may also include additional comments for the author, including concerns about dual publication, research ethics, or publication ethics. (Please upload your review as an attachment if it exceeds 20,000 characters)

Reviewer #1: Abstract
Under Methods

Reviewer#1 comments: Line 6: Authors need to add the word cross to Sectional study

**Authors’ response**: Authors’ responses: Thank you so much. We appreciated your valuable comments. We have corrected in the revised manuscript

Reviewer#1 comment: Line 8: Authors need to change Data “was” to were
**Authors’ response:** Thank you a lot for your substantial effort. We have corrected n the revised manuscript

Reviewer#1 comment: Line 9: Authors need to change was to were after food frequency questionnaires

**Authors’ response**: Thank you so much. We appreciated your comments. We have corrected in the revised manuscript
Introduction
Paragraph one
Reviewer#1 comment: Authors need to get more updated statistics since the figure of 3.5 million is from a 2008 reference. Consider citing (Sserwanja, Q, Kawuki, J, Mutisya, LM, et al. Underweight and associated factors among lactating women in Uganda: Evidence from the Uganda demographic health survey 2016. Health Sci Rep. 2021; 4:e356. https://doi.org/10.1002/hsr2.356) with updated statistics

**Authors’ response**: Thank you a lot for your substantial effort. We have updated statistics in the revised manuscript:

Paragraph four
Reviewer#1comment: Line 2: Authors need to remove s between and & nutritional status

**Authors’ response**: Thank you so much. We appreciated your comments. We have corrected in the revised manuscript
Methods

Paragraph 3: Source population and study population

Reviewer#1 comment: Revise grammar of the first and second sentences
**Authors’ response**: Thank you so much. We appreciated your comments. We have corrected grammar in the revised manuscript
Reviewer#1 comment: Paragraph 4: Inclusion and exclusion criteria
Authors need to revise the grammar of the first and second sentences

**Authors’ response**: Thank you a lot for your substantial effort. We have corrected grammar in the revised manuscript
Paragraph 6
Reviewer#1 comment: Authors need to change data was to data were in the first sentence

**Authors’ response**: Thank you so much. We appreciated your comments. We have corrected grammar in the revised manuscript

Reviewer#1 comment: Authors need to elaborate to the readers why they choose MUAC as a method of assessing undernutrition and why not other methods

**Authors’ response**: Thank you so much. We appreciated your comments. We have clearly described why we use MUAC as a method of assessing under-nutrition in the revised manuscript. We have stated as the following

“The body mass index (BMI, kg/m2) is currently the gold standard for measuring body fatness. However, pregnancy-associated weight gain and oedema, as well as late booking into antenatal care in our population setting, causes us to question the reliability of using the BMI to assess body fat or nutritional status in pregnancy. The MUAC is a much simpler anthropometric measure than the BMI, as its use eliminates the need for expensive equipment, such as height charts and scales, and the need for calculations. It is also much easier to perform on a patient who is acutely unwell, bed bound or sedentary. Another important advantage of using MUAC is that there is minimal change in the MUAC during pregnancy, so it may be a better indicator of pre-pregnancy body fat and nutrition than the BMI”

Paragraph 8: Analysis
Reviewer#1 comment: Authors need to clarify if by normal being coded as 0, did this include overweight and obese women, or they were unable to measure this?

**Authors’ response**: Thank you a lot for your substantial effort. We have used the cut of point recommended by WHO.

**Authors’ response**: Thank you a lot for your substantial effort. We have corrected in the revised manuscript.

Dear reviewer in this revised manuscript we have revised the model of analysis from logistic regression to, Poisson regression analysis model with robust variance estimate for analysis. Poisson regression is used for count outcome variable. However, Poisson regression analysis model with robust variance estimate is used for binary outcome. In our case we have tried to fit models like logistic regression, Binomial and Poisson regression analysis model with robust variance estimate. Even though the three models will be used for binary outcome they have their own limitation. First I fitted with logistic regression, Then Binomial Model and finally Poisson regression analysis model with robust variance estimate. Poisson regression analysis model with robust variance estimate gave me a narrow confidence interval and I choose this one. Regarding the variable we have checked the model fitness with AIC and BIC. There are many variables fitted to multivariate model, but during model fitness selection with stepwise a few variables are removed from the model and we checked the two model.

We had also used this model. For our previous polished paper was

Here below the stata command and output

Reviewer#1 comment: Were descriptives done? If yes, authors need to mention this.

Authors’ response: Thank you so much. We appreciated your comments. We have to do descriptive analysis of socio-demographic characteristics and anthropometric and nutritional status of respondents

RESULTS

Reviewer#1 comment:

Paragraph 1:
Authors need to explain why they chose to have cannot read and write, informal and formal education as the sub-categories.

What was the cut off for formal education? Was a primary school candidate the same as a tertiary institute candidate? How was informal education defined?Why was the classification of husband education different from that of the woman? Under husband education, how different was can read and write from high school and above?

**Authors’ response:** Thank you a lot for your substantial effort. We have revised the women’s educational sub-categories as “cannot read and write, can read and write and formal education “. We had collected all the educational level of the respondent. However most of the respondents cannot read and write. Thus we put sub-categories into three. Formal education is defined as the educational status with minim of grade one in school. Those women who had adult functional education were label as “can read and write”

Moreover we had collected all the educational level of the husbands. However since the information on their educational status was majorly distributed with in sub-categories of “cannot read and write, can read and write 1-8 Grade 1-8 and Grade 9 and above”. Thus we put the available information within sub-categories.

Reviewer#1 comment:

Note: Authors need to describe how these were agreed upon in the methods section
Gravidity and Parity are the almost same. Why did the authors add both?

**Authors’ response**: Thank you so much. We appreciated your comments. We have used the stage of pregnancy in the revised manuscript. :

Discussion
Paragraph 3:
Reviewer#1 comment : Revise grammar of first sentence

**Authors’ response**: Thank you so much. We appreciated your comments. We have corrected grammar in the revised manuscript

Conclusion
Reviewer#1 comment: Revise grammar of second sentence

**Authors’ response**: Thank you so much. We appreciated your comments We have corrected grammar in the revised manuscript
References
Reviewer#1 comment : Reference 8: Change to (Sserwanja, Q., Mukunya, D., Habumugisha, T. et al. Factors associated with undernutrition among 20 to 49 year old women in Uganda: a secondary analysis of the Uganda demographic health survey 2016. BMC Public Health 20, 1644 (2020). https://doi.org/10.1186/s12889-020-09775-2). It is the same paper but the one cited was a thesis and this is the published article
Kindly revise all the other references to ensure that they are complete with journal name, issue/volume and page number (OR as per journal’s requirement)

**Authors’ response**: Thank you so much. We appreciated your comments we have tied to correct the references in the revised manuscript

“The sample size was determined using single and double population proportion formulas with their corresponding assumption, and the largest sample size was considered. As such, the sample was computed using the single population proportion formula with the following assumptions: 95% confidence interval, the prevalence of undernutrition among pregnant women Gumay District, (44.9%) (16), 5% marginal error, and 10% non-response rate; the final computed sample size was 419. However, since this study was part of a larger longitudinal study (a prospective cohort study aimed to assess neonates’ birth weight and the association with maternal iron status), the same 475 pregnant women were included. A detailed description has been given elsewhere in the previous papers (21, 23, 24).

This is one of study par longitudinal large study conducted for the purpose of the corresponding author’ dissertation from which five papers are published for for your information articles listed below

I. Fite MB, Tura AK, Yadeta TA, Oljira L, Roba KT. Prevalence and determinants of dietary practices among pregnant women in eastern Ethiopia. BMC nutrition. 2022 Dec;8(1):1-10.

# II. Fite MB, Tura AK, Yadeta TA, Oljira L, Roba KT (2022) Consumption of animal source food and associated factors among pregnant women in eastern Ethiopia: A community-based study. PLoS ONE 17(6): e0270250. <https://doi.org/> 10.1371/journal.pone.027025

# III. Fite MB, Tura AK, Yadeta TA, Oljira L, Roba KT. Factors associated with food consumption score among pregnant women in Eastern Ethiopia: a community-based study. Journal of Health, Population and Nutrition. 2022 Dec;41(1):1-9.

# IV. Fite MB, Tura AK, Yadeta TA, Oljira L, Roba KT (2022) Factors associated with undernutrition among pregnant women in eastern Ethiopia: A community-based study. PLoS ONE (Revision under-review)

# V. Fite MB, Tura AK, Yadeta TA, Oljira L, Roba KT. Beyond Hemoglobin: Uncovering iron deficiency and iron deficiency anemia using serum ferritin concentration in eastern Ethiopia: A community-based study. BMC nutrition. (Revision under-review )

VI. Fite MB, Tura AK, Yadeta TA, Oljira L, Roba KT. Prevalence, predictors of low birth weight and its association with maternal iron status using serum ferritin concentration in rural Eastern Ethiopia: Prospective cohort study BMC nutrition. (Accepted for publication)

Reviewer #2: comments
Manuscript Id: PONE-D-21-17941
Title: Determinants of Under-nutrition Among Pregnant Women in Haramaya District, Eastern Ethiopia

Comments
*While the title and the objectives talk about determinants of under-nutrition, the authors used a cross sectional study and reported only factors associated with under-nutrition, not the determinants. The authors should have used case control study. You have to modify the title as “Factors associated with under-nutrition among Pregnant Women in Haramaya District, eastern Ethiopia”, or otherwise the evidence presented doesn’t match your title and objectives.

*I suggest to write “Among” as ‘among’ and “Eastern” as ‘eastern’ in your revised title
You used different font styles for similar headings and sub-headings. Please check and revise it.

**Authors’ response**: Thank you a lot for your substantial effort. We have corrected in the revised manuscript

We have tied to revise title as “Factors associated with undernutrition among pregnant women in eastern Ethiopia: A community-based study”

Reviewer #2: Comments: *The manuscript is full of grammatical and editorial errors. Please go through it line by line (thoroughly) and revise it, better checked by language professionals. Some of such errors are indicated below.

**Authors’ response**: Thank you so much. We appreciated your comments. We have corrected grammar in the revised manuscript
Abstract
Reviewer #2: Comments: Methods: - sectional study design was….

**Authors’ response**: Thank you so much. We appreciated your comments. We have corrected in the revised manuscript

Reviewer #2: Comments:
*But, what is sectional study design? Please make it clear.
- Data was collected with interviewers-administered questionnaires by well-trained health professionals.
*Make it data were collected with…. And this is also same under method section.
*well trained health professionals….who are these, HDSS staff? What do you mean by well trained? Please clearly indicate what their profession is, their level of education is and how their training was for this data collection. Describe these details under your methods section.

**Authors’ response**: Thank you a lot for your substantial effort. We have corrected in the revised manuscript.

Dear reviewer in this revised manuscript we have revised the model of analysis from logistic regression to, Poisson regression analysis model with robust variance estimate for analysis. Poisson regression is used for count outcome variable. However, Poisson regression analysis model with robust variance estimate is used for binary outcome. In our case we have tried to fit models like logistic regression, Binomial and Poisson regression analysis model with robust variance estimate. Even though the three models will be used for binary outcome they have their own limitation. First I fitted with logistic regression, Then Binomial Model and finally Poisson regression analysis model with robust variance estimate. Poisson regression analysis model with robust variance estimate gave me a narrow confidence interval and I choose this one. Regarding the variable we have checked the model fitness with AIC and BIC. There are many variables fitted to multivariate model, but during model fitness selection with stepwise a few variables are removed from the model and we checked the two model.

We had also used this model. For our previous polished paper was

Here below the stata command and output

Results:

Reviewer #2: Comments: line 2- “Under-nutrition” is written in different font from others.
-Line 3 Breakfast should be written as ‘breakfast’

**Authors’ response**: Thank you a lot for your substantial effort. We have corrected in the revised manuscript

Reviewer #2: Comments: Conclusion: -The the present study“
*What is “the the”? Please edit it.

**Authors’ response**: Thank you so much.. We have corrected in the revised manuscript

Reviewer #2: Comments:
- High prevalence “was observed on” women who “reported chat less than four times”
* was observed on should be written as “was found among”
*reported chat less than four times should be “ reported to chew Khat less than four times”

**Authors’ response**: Thank you a lot for your substantial effort. We have corrected in the revised manuscript

Reviewer #2: Comments:
-Nutrition policy, programs and interventions….
* This is too general and ambiguous conclusion. Based on your findings specify to the point and indicate which policy, program and intervention should do what to improve which problem.

**Authors’ response**: Thank you so much.. We have tied to revise in the revised manuscript

Introduction
Reviewer #2: Comments

*This section is not well written since doesn’t indicate the picture of maternal under-nutrition during pregnancy from global, regional, national and local contexts.
* The literatures are not well searched, and hence the introduction is too shallow. So many other related articles were missed.

**Authors’ response**: Thank you a lot for your substantial effort. We have corrected in the revised manuscript

Reviewer #2: Comments:
*What is the importance of this study since the factors identified were already well known as you indicated under paragraph three? What is special now in this study? Can’t we use studies from other settings in Ethiopia for eastern Ethiopian regions?

**Authors’ response**: Thank you a lot for your substantial effort. Since this is study is community based study it could provide the real information regarding nutritional status of pregnant women. Additionally we have carried out the assessment of anemia using hemoglobin level.

Reviewer #2: Comments:
-Line 2 ….affecting the healthy of women both in developed and developing countries, where, more than…
* write it as ‘affecting health of women both in developed and developing countries where more than…
Line 3…Moreover, under-nutrition occurred due to the…
*Write it as ‘Moreover, under-nutrition occurs due to the….
-Paragraph 4 line 2 ….and s nutritional status among
* re-write it as … and nutritional status among

**Authors’ response**: Thank you a lot for your substantial effort. We have corrected in the revised manuscript
Methods

Reviewer #2: Comments: *Chat has to be written as ‘Khat’, revise it accordingly throughout the document

**Authors’ response**: Thank you a lot for your substantial concern. We have corrected in the revised manuscript
Reviewer #2: Comments: *You excluded all pregnant women with reported acute and chronic illnesses. How trustable this reported illness is? And what number or how many percent of the women were excluded with these criteria?

**Authors’ response**: Thank you a lot for your substantial concern. We excluded chronic illnesses based on respondent’s information only.

Reviewer #2: Comments:
-The sample size required and adequate for estimating the determinants of dietary practices of pregnant women was computed….
*Are you calculating for determinants of dietary practice or under nutrition? Be consistent, clear and to the point in line with your objectives.
*For sample size determination you just stated of using single and double population proportions. Would you indicate the double proportion formula and calculations used? *How many variables were used to try calculating your sample size using double proportion formula? Please indicate these as well.

**Authors’ response**: Thank you a lot for your substantial concern. We have tried to revise the sample size calculation in the revised manuscript. Here below is the sample size calculation procures.

Since this study was part of the larger longitudinal study which obtained birth outcome information of pregnant women we took the larger sample size and response rate for this study is corrected in the revised manuscript. Here is the way we calculated the sample size for the larger study.

“The sample size was determined using single and double population proportion formulas with their corresponding assumption, and the largest sample size was considered. As such, the sample was computed using the single population proportion formula with the following assumptions: 95% confidence interval, the prevalence of undernutrition among pregnant women Gumay District, (44.9%) (16), 5% marginal error, and 10% non-response rate; the final computed sample size was 419. However, since this study was part of a larger longitudinal study (a prospective cohort study aimed to assess neonates’ birth weight and the association with maternal iron status), the same 475 pregnant women were included. A detailed description has been given elsewhere in the previous papers (21, 23, 24).

This is one of study par longitudinal large study conducted for the purpose of the corresponding author’ dissertation from which five papers are published for for your information articles listed below

I. Fite MB, Tura AK, Yadeta TA, Oljira L, Roba KT. Prevalence and determinants of dietary practices among pregnant women in eastern Ethiopia. BMC nutrition. 2022 Dec;8(1):1-10.

# II. Fite MB, Tura AK, Yadeta TA, Oljira L, Roba KT (2022) Consumption of animal source food and associated factors among pregnant women in eastern Ethiopia: A community-based study. PLoS ONE 17(6): e0270250. <https://doi.org/> 10.1371/journal.pone.027025

# III. Fite MB, Tura AK, Yadeta TA, Oljira L, Roba KT. Factors associated with food consumption score among pregnant women in Eastern Ethiopia: a community-based study. Journal of Health, Population and Nutrition. 2022 Dec;41(1):1-9.

# IV. Fite MB, Tura AK, Yadeta TA, Oljira L, Roba KT (2022) Factors associated with undernutrition among pregnant women in eastern Ethiopia: A community-based study. PLoS ONE (Revision under-review)

# V. Fite MB, Tura AK, Yadeta TA, Oljira L, Roba KT. Beyond Hemoglobin: Uncovering iron deficiency and iron deficiency anemia using serum ferritin concentration in eastern Ethiopia: A community-based study. BMC nutrition. (Revision under-review )

VI. Fite MB, Tura AK, Yadeta TA, Oljira L, Roba KT. Prevalence, predictors of low birth weight and its association with maternal iron status using serum ferritin concentration in rural Eastern Ethiopia: Prospective cohort study BMC nutrition. (Accepted for publication)

Reviewer #2: Comments:

* You described the role of agro-ecolocolgical difference on under-nutrition, but used study from Gumay district for sample size determination. Why? Any effect it might pose

**Authors’ response**: Thank you a lot for your substantial concern. We have used different studies which are similar agro-ecolocolgical characteristics with our study area but we are obligated to use the larger sample size.
Reviewer #2: Comments: * Also mention names of those kebeles (with their total sample allocated), you used under the DSS and techniques followed using a simple diagrammatic presentation.

**Authors’ response**: Thank you a lot for your substantial effort. We have tried to mention the name of kebeles (with their total sample allocated). Here below is a simple diagrammatic presentation

Haramaya HDSS with 8 kebeles and 1513 pregnant women

Kuro=234

Ifa orom=206

Bacaqe=81

Damota=118

Qerensa=192

Nagya=195

Finkile=96

Bif.Gada=255

Amuma=166

S

Sample of each kebele will be determined proportionally from pregnant women that exist at study period

Kuro=73

Ifa orom=65

Damota=37

Bacaqe=25

Qerensa=60

Nageya=61

Finkile=31

Bif.Gada=71

Amuma=52

SRS technique will be using to prepare sampling frame from house-to-house visit

475

**Figure1: Schematic representation of sampling procedure for selection of pregnant women in Haramaya district, April 2021**

-After translation, it’s the consistency * write it as “its consistency”
* Indicate/cite literatures you used for developing your questionnaire

Reviewer #2: Comments:
- Hemoglobin analysis was carried out in the health post located in each Kebele by laboratory technologists.
* How was this practical? You were collecting data home to home but hemoglobin at health center? Were you calling each women to health center? What is the need to go to health center since you used Hemocue?
-Hemoglobin level was adjusted for altitude before the data were entered.
*How was the adjustment done- any formula? What about adjustment for other factors- did you considered any? Indicate how you classified anemia under analysis part.

**Authors’ response**: Thank you a lot for your substantial effort. This stud is the part of larger study in which we also assess the micronutrient level of pregnant women and birth outcome. Sorry for the error we have done testing at each study site. The adjustment of hemoglobin, WHO recommends correcting the cut-off point of hemoglobin to define anemia in high-altitude (HA) populations . Accordingly the adjustment was performed. Moreover anemia was defined as a Hemoglobin level of < 11.0 g/dl during the first or third trimester or <10.5 g/dl during the second

Altitude adjustments to measured haemoglobin concentrations

| Altitude (metres above sea level) | Measured haemoglobin adjustment (g/l) |
| --- | --- |
| < 1000 | 0 |
| 1000 | -2 |
| 1500 | -5 |
| 2000 | -8 |
| 2500 | -13 |
| 3000 | -18 |
| 3500 | -27 |
| 4000 | -35 |
| 4500 | -45 |

Reference

WHO. Haemoglobin concentrations for the diagnosis of anaemia and assessment of severity. Vitamin and Mineral Nutrition Information System. Geneva, World Health Organization, 2011 (WHO/NMH/NHD/MNM/11.1) (<http://www.who.int/vmnis/indicators/haemoglobin>.

Reviewer #2: Comments:
* standard operating procedures (SOPs)….indicate these under the procedures….only SOP you used.
* How was anthropometric measurement standardized? Any TEM (technical error of measurements) done? Try to answer this in relation to the data collectors training, profession and how were they trained as raised above?

**Authors’ response**: Thank you a lot for your substantial effort. We have tied to indicate the procedures in the revised manuscript. Here below is the description stated.

In addition, mid-upper arm circumference (MUAC) and maternal height measurements were taken.  The nutritional status of the pregnant women was measured with non-stretchable MUAC tape and the reading value was taken to the nearest 0.1-cm. All measurements were performed threefold and the average value of two concordant readings was considered as the ultimate value. Pregnant women with average MUAC measurements of less than 23 cm were categorized as having “undernutrition” otherwise normal (25, 26). The questionnaire was initially prepared in English and translated to the local language (Afan Oromo) by individuals with good command of both languages. It was also pre-tested on 10% of the samples in Kersa District before actual implementation. Women's hemoglobin concentration (in g/dL) was measured at each study site by well-trained medical technologists using HemoCue® Hb 301 system, according to the manufacturer's instructions (HemoCue AB Ängelholm Sweden) which is a gold standard for fieldwork. A prick was done on the tip of the middle finger after the site was cleaned with disinfectant. The first drop of blood was cleaned off and the second drop was collected to fill the microcuvette which is then placed in the cuvette holder of the device for measuring hemoglobin concentration. Hemoglobin values were adjusted for altitude as per the Center for Disease Prevention and Control (CDC) recommendation (27). Reviewer #2: Comments: * under analysis- you still stated “to identify determinants of under-nutrition”….you rather should say factors associated with under-nutrition, not determinants…..And you may also need to re-consider your method of analysis appropriate to this…..logistic regression is one.

* What about those women under age regarding consent to participate? You had 24 women under the age of 18 years.

**Authors’ response**: Thank you a lot for your substantial effort. We have tried to indicate the about the consents of below 18 in ethical consideration part as “Written informed consent to participate was obtained from participants and legally authorized representatives "of minors below 16 years of age and illiterates" and their privacy and confidentiality were maintained. We have also revised the title as “Factors associated with Under-nutrition among pregnant women in eastern Ethiopia: A community-based study” .

Results
Reviewer #2: Comments: *How do you see 100% response rate in a community based study like yours?

Thank you a lot for your substantial concern. We have tied to revise the method used for calculating the sample size in revised manuscript. Accordingly this a total of 475 pregnant women were eligible, and 448 consented, making a response rate of 94.3%

Reviewer #2: Comments:
*Table 1- would you classify and present women’s education similar to husbands’ case?
*How do you see the wealth index vs women’s and husband’s occupation? Mismatch?
* Table 2: nutritional status ….write it as under-nutrition and normal….check typos errors in the table and also do not mix acute…make it consistent from title to conclusion.
* Chat chewing….make it “Khat”

**Authors’ response**: Thank you a lot for your substantial effort. We have tried to correct in the revised manuscript.

Reviewer #2: Comments:

* Table 3…factors determinants of under-nutrition…..vs the sub-title factors associated with??? And also vs manuscript title, check it again. I suggest this analysis method to be changed as indicated above.
* And also try to present some of your data with other data presentation techniques than table…
**Authors’ response**: Thank you a lot for your substantial effort. We have tried to correct in the revised manuscript.

Discussion
Reviewer #2: Comments *This section is very shallow. You are not supposed just to say similarity and difference ,rather explain what the public health implication of each finding is and interprete.
E.g. You put “This might be the gio-ecological variation of study set up, methodology used, where some of them were conducted at facility level which does not really show the real magnitude of the problem as of community level”.
*You have to tell the readers how geo-ecological variation matters, what method difference brought difference in the findings,,,,etc?

**Authors’ response**: Thank you a lot for your substantial effort. We have tried to correct the discussion in the revised manuscript.

Reviewer #2: Comments *Re-look at the strengths and limitations you put…a lot of limitations with less strengths.

**Authors’ response**: Thank you a lot for your substantial effort. We have tried to correct the discussion in the revised manuscript.

Conclusion

Reviewer #2: Comments

*How would you comment on wealth status and high under-nutrition in your findings? *Check comments to the comments to conclusion under abstract

* And re-look at your recommendations regarding anemia and Iron folic acid supplementation.

**Authors’ response**: Thank you a lot for your substantial effort. We have tried to correct the conclusion in the revised manuscript.
