# Supplementary material for: Factors associated with undernutrition among pregnant women in Haramaya district, Eastern Ethiopia: A community-based study
Source: PLoS One. 2023 Mar 9;18(3):e0282641. doi: 10.1371/journal.pone.0282641 (PMC9997975; doi:10.1371/journal.pone.0282641)
Supplement: Attachment — Submitted filename: Authors response to reviewers comments MUAC.docx [file pone.0282641.s003.docx]

PLOS ONE Decision: Revision required [PONE-D-21-17941R1] - [EMID:457528c049d4a475]

PONE-D-21-17941R1

Factors associated with undernutrition among pregnant women in Haramaya District, Eastern Ethiopia: A community-based study

PLOS ONE

First of all we would like to acknowledge you for your constructive and valuable comments and helpful
suggestions that helped us to improve and enrich our manuscript. Here under following editor’s comment and each reviewer’s we have pointed out how authors incorporated your valuable comments, suggestions and concerns one by one. On the following pages, you will find our response to the editor and reviewer comments. On behalf of my co-authors, I thank you for your consideration of this re-submission.

Meseret Belete Fite, meseretphd2014@gmail.com (correspondence author)

Editor comments and authors’ response

Thank you for submitting your manuscript to PLOS ONE. After careful consideration, we feel that it has merit but does not fully meet PLOS ONE’s publication criteria as it currently stands. Therefore, we invite you to submit a revised version of the manuscript that addresses the points raised during the

review process.

Please submit your revised manuscript by Nov 21 2022 11:59PM. If you will need more time than this to complete your revisions, please reply to this message or contact the journal office at [plosone@plos.org](mailto:plosone@plos.org). When you're ready to submit your revision, log on to <https://www.editorialmanager.com/pone/> and select the 'Submissions Needing Revision' folder to locate your manuscript file.

Please include the following items when submitting your revised manuscript:

- A rebuttal letter that responds to each point raised by the academic editor and reviewer(s). You should upload this letter as a separate file labeled 'Response to Reviewers'.
- A marked-up copy of your manuscript that highlights changes made to the original version. You should upload this as a separate file labeled 'Revised Manuscript with Track Changes'.
- An unmarked version of your revised paper without tracked changes. You should upload this as a separate file labeled 'Manuscript'.

If you would like to make changes to your financial disclosure, please include your updated statement in your cover letter. Guidelines for resubmitting your figure files are available below the reviewer comments at the end of this letter.

If applicable, we recommend that you deposit your laboratory protocols in [protocols.io](http://protocols.io/) to enhance the reproducibility of your results. Protocols.io assigns your protocol its own identifier (DOI) so that it can be cited independently in the future. For instructions see: <https://journals.plos.org/plosone/s/submission-guidelines#loc-laboratory-protocols>. Additionally, PLOS ONE offers an option for publishing peer-reviewed Lab Protocol articles, which describe protocols hosted on [protocols.io](http://protocols.io/). Read more information on sharing protocols at <https://plos.org/protocols?utm_medium=editorial-email&utm_source=authorletters&utm_campaign=protocols>.

We look forward to receiving your revised manuscript.

Kind regards,

Jianhong Zhou

Staff Editor

PLOS ONE

Journal Requirements:

Please review your reference list to ensure that it is complete and correct. If you have cited papers that have been retracted, please include the rationale for doing so in the manuscript text, or remove these references and replace them with relevant current references. Any changes to the reference list should be mentioned in the rebuttal letter that accompanies your revised manuscript. If you need to cite a retracted article, indicate the article’s retracted status in the References list and also include a citation and full reference for the retraction notice.

Reviewers' comments:

Reviewer's Responses to Questions

**Comments to the Author**

1. If the authors have adequately addressed your comments raised in a previous round of review and you feel that this manuscript is now acceptable for publication, you may indicate that here to bypass the “Comments to the Author” section, enter your conflict of interest statement in the “Confidential to Editor” section, and submit your "Accept" recommendation.

Reviewer #1: (No Response)

2. Is the manuscript technically sound, and do the data support the conclusions?

The manuscript must describe a technically sound piece of scientific research with data that supports the conclusions. Experiments must have been conducted rigorously, with appropriate controls, replication, and sample sizes. The conclusions must be drawn appropriately based on the data presented.

Reviewer #1: Yes

3. Has the statistical analysis been performed appropriately and rigorously?

Reviewer #1: Yes

4. Have the authors made all data underlying the findings in their manuscript fully available?

The [PLOS Data policy](http://www.plosone.org/static/policies.action#sharing) requires authors to make all data underlying the findings described in their manuscript fully available without restriction, with rare exception (please refer to the Data Availability Statement in the manuscript PDF file). The data should be provided as part of the manuscript or its supporting information, or deposited to a public repository. For example, in addition to summary statistics, the data points behind means, medians and variance measures should be available. If there are restrictions on publicly sharing data—e.g. participant privacy or use of data from a third party—those must be specified.

Reviewer #1: Yes

5. Is the manuscript presented in an intelligible fashion and written in standard English?

PLOS ONE does not copyedit accepted manuscripts, so the language in submitted articles must be clear, correct, and unambiguous. Any typographical or grammatical errors should be corrected at revision, so please note any specific errors here.

Reviewer #1: Yes

**Reviewer's comments and authors’ response**

6. Review Comments to the Author

Please use the space provided to explain your answers to the questions above. You may also include additional comments for the author, including concerns about dual publication, research ethics, or publication ethics. (Please upload your review as an attachment if it exceeds 20,000 characters)

Reviewer #1: Thank you so much for working on the comments.

Reviewer#1 comments

General comments

Kindly line number the manuscript and in the responses, add pages and line numbers for easier follow up

**Authors’ response**: Thank you so much. We appreciated your comments. We have added pages and line numbers in the revised manuscript

Reviewer#1 comments

In the methods, kindly add a section describing the independent variables and how they were classified

**Authors’ response**: Thank you so much. We appreciated your comments. We have corrected in the revised manuscript
Specific comments

Kindly see my comments on your responses:

Your response

Reviewer#1 comment: Authors need to elaborate to the readers why they choose MUAC as a method of assessing undernutrition and why not other methods
Authors’ response: Thank you so much. We appreciated your comments. We have clearly described why we use MUAC as a method of assessing under-nutrition in the revised manuscript. We have stated as the following“The body mass index (BMI, kg/m2) is currently the gold standard for measuring body fatness. However, pregnancy-associated weight gain and oedema, as well as late booking into antenatal care in our population setting, causes us to question the reliability of using the BMI to assess body fat or nutritional status in pregnancy. The MUAC is a much simpler anthropometric measure than the BMI, as its use eliminates the need for expensive equipment, such as height charts and scales, and the need for calculations. It is also much easier to perform on a patient who is acutely unwell, bed bound or sedentary. Another important advantage of using MUAC is that there is minimal change in the MUAC during pregnancy, so it may be a better indicator of pre-pregnancy body fat and nutrition than the BMI”

Reviewer#1 comments

My comment:

I seem not to see this. Kindly line show which pages this information is.
Your response

Reviewer#1 comment: Were descriptives done? If yes, authors need to mention this.
Authors’ response: Thank you so much. We appreciated your comments. We have to do descriptive analysis of socio-demographic characteristics and anthropometric and nutritional status of respondents

Reviewer#1 comments

My comment

Kindly add this in the data processing and analysis section

**Authors’ response**: Thank you so much. We appreciated your comments. We have corrected in the revised manuscript. The blow statements is stated in methods section (under operational definition” as :

“ Frequencies, percentages, summary measures and tables were used to describe and present the descriptive). Frequencies, percentages, summary measures and tables were used to describe and present the descriptive information of respondents. The MUAC is a much simpler anthropometric measure than the BMI, as its use eliminates the need for expensive equipment, such as height charts and scales, and the need for calculations. It is also much easier to perform on a patient who is acutely unwell, bed bound or sedentary. Another important advantage of using MUAC is that there is minimal change in the MUAC during pregnancy, so it may be a better indicator of pre-pregnancy body fat and nutrition than the BMI.”

Your response

Authors need to explain why they chose to have cannot read and write, informal and formal education as the sub-categories.What was the cut off for formal education? Was a primary school candidate the same as a tertiary institute candidate? How was informal education defined?Why was the classification of husband education different from that of the woman? Under husband education, how different was can read and write from high school and above?

Authors’ response: Thank you a lot for your substantial effort. We have revised the women’s educational sub-categories as “cannot read and write, can read and write and formal education “. We had collected all the educational level of the respondent. However most of the respondents cannot read and write. Thus we put sub-categories into three. Formal education is defined as the educational status with minim of grade one in school. Those women who had adult functional education were label as “can read and write”
Moreover we had collected all the educational level of the husbands. However since the information on their educational status was majorly distributed with in sub-categories of “cannot read and write, can read and write 1-8 Grade 1-8 and Grade 9 and above”. Thus we put the available information within sub-categories

Reviewer#1 comments

My comment:

1. Did you base on the national guidelines to say grade 1 is formal education for women?

**Authors’ response**: Thank you a lot for your substantial effort for clarification. According to the Ethiopian education, policy people who had at least grade one education level are categorized as having “formal educational level”. However, those could able to read and write due to different reason are labeled as having “informal education”. Whereas people who could not able to read and write are categorized as “illiterate”. In the revised manuscript, we have tried to correct the women educational level. Even though we had described the women education level in three category in table 1 “ cannot read , write and can read and write and formal” we have tried to merge those cannot read and write and can read and write (informal) in table 3 for analysis purpose. Thus, we got the new variables with two responses cannot read and write and can read and write (informal) and grade one and above (Formal education) was significant at bi-variable analysis.

Reviewer#1 comments

2. Why did you remove formal education sub-category for women and only considered can read and write and cannot read and write in Table 3?

**Authors’ response**: Thank you so much. We appreciated your comments. We have tied to correct in the revised manuscript. We have included formal education sub-category

Reviewer#1 comments

3. Kindly add this categorization in the methods section

**Authors’ response**: Authors’ responses: Thank you so much. We appreciated your valuable comments. We have added in the revised manuscript. The blow statements is stated in methods section (under operational definition” as :

**“Educational status:** Respondents who had grade at least grade one education level were labeled as“ formal”, whereas respondents those could able to read or write sentences were categorized as” Informal education”

Your response

Reviewer#1 comment:

Note: Authors need to describe how these were agreed upon in the methods section
Gravidity and Parity are the almost same. Why did the authors add both?
Authors’ response: Thank you so much. We appreciated your comments. We have used the stage of pregnancy in the revised manuscript. :

Reviewer#1 comments

My comment
Its good to look at the stage of pregnancy but also good to maintain parity/number of children/gravidity. Any particular reason why you decided to remove this?

**Authors’ response**: Thank you so much. We appreciated your request for clarification. Regarding the reason the why we removed parity/number of children/gravidity was, these variables were not candidate in bi-variable analysis of the new model we used.

7. PLOS authors have the option to publish the peer review history of their article ([what does this mean?](https://journals.plos.org/plosone/s/editorial-and-peer-review-process#loc-peer-review-history)). If published, this will include your full peer review and any attached files.


If you choose “no”, your identity will remain anonymous but your review may still be made public.


**Do you want your identity to be public for this peer review?** For information about this choice, including consent withdrawal, please see our [Privacy Policy](https://www.plos.org/privacy-policy).

Reviewer #1: **Yes:** sserwanja Quraish

[NOTE: If reviewer comments were submitted as an attachment file, they will be attached to this email and accessible via the submission site. Please log into your account, locate the manuscript record, and check for the action link "View Attachments". If this link does not appear, there are no attachment files.]

While revising your submission, please upload your figure files to the Preflight Analysis and Conversion Engine (PACE) digital diagnostic tool, <https://pacev2.apexcovantage.com/>. PACE helps ensure that figures meet PLOS requirements. To use PACE, you must first register as a user. Registration is free. Then, login and navigate to the UPLOAD tab, where you will find detailed instructions on how to use the tool. If you encounter any issues or have any questions when using PACE, please email PLOS at [figures@plos.org](mailto:figures@plos.org). Please note that Supporting Information files do not need this step.
